# Editorial: Role of RNA in Molecular Diagnostics of Cancer

**DOI:** 10.3389/fgene.2020.00435

**Published:** 2020-05-06

**Authors:** William C. S. Cho, Lawrence W. C. Chan, Cesar S. C. Wong

**Affiliations:** ^1^Department of Clinical Oncology, Queen Elizabeth Hospital, Hong Kong, China; ^2^Department of Health Technology and Informatics, Faculty of Health and Social Sciences, Hong kong Polytechnic University, Hong Kong, China

**Keywords:** cancer, circulating cell-free RNA, liquid biopsy, molecular diagnostics, RNA

The central dogma of molecular biology is an explanation of the flow of genetic information within a biological system. RNA is a dynamic bridge between the blueprint of life, DNA and the executor, protein. There is a broad spectrum of RNA, playing key roles in the living organisms, from physiological to pathophysiological processes.

RNA is essential for various biological roles, including coding, decoding, regulation, and expression of genes. In the world of RNA, about 2% is coding RNAs, while non-coding RNAs (ncRNAs) make up the majority (98%) of the transcriptomes. ncRNAs are RNA molecules transcribed from DNA but not translated into proteins. They regulate gene expression at the transcriptional and post-transcriptional levels. There are many types of ncRNAs, including tRNAs, rRNAs, long ncRNAs, and small RNAs (such as microRNAs, siRNAs, piRNAs, circRNA, snoRNAs, snRNAs, exRNAs). These molecules have fundamental roles in cells and many are stable in body fluids as extracellular RNAs. This Research Topic (RT) opens a platform for the update of the scientific development of RNA in molecular diagnostics of cancer.

MicroRNAs (miRNAs) are a class of ncRNAs that can be involved in the regulation of gene expression in cancer. Tian et al. found that miR-486-5p was significantly downregulated in non-small cell lung cancer (NSCLC) samples. PIK3R1 gene was confirmed to be the target of miR-486-5p. Overexpressed miR-486-5p effectively inhibited cell proliferation, invasion and successfully induced apoptosis *in vitro*. PIK3R1 also involved in the suppression of miR-486-5p on cell growth. They concluded that miR-486-5p might act as a tumor suppressor contributing to the progression of NSCLC, and miR-486-5p might be a diagnostic/prognostic biomarker and a potential therapeutic target for lung cancer. On the other hand, Zhang et al. identified the association of miR-153 with bladder cancer (BCA) prognosis using The Cancer Genome Atlas (TCGA) dataset. They found that miR-153 expression was downregulated in BCA tissues and cell lines. Reduced miR-153 expression was associated with poor prognosis of advanced stage patients. Furthermore, miR-153 expression inhibited BCA cell growth by promoting tumor cell apoptosis, migration, invasion, and epithelial–mesenchymal transition (EMT) *in vitro* and tumor xenograft growth *in vivo*, it could also suppress human umbilical vein endothelial cell and chicken chorioallantoic membrane angiogenesis. In addition, they found that miR-153 targeted IDO1 expression and inhibited BCA cell tryptophan metabolism through inhibiting IL6/STAT3/VEGF signaling. They thus concluded that miR-153 exerted anti-tumor activity in BCA by targeting IDO1 expression, miR-153 might serve as a novel therapeutic target for BCA patients.

Emerging evidence is accumulating that tumor cells release substantial amounts of RNA into the bloodstream, which strongly resist RNases in the blood. This liquid biopsy contains circulating RNAs which are usually packed in extracellular vesicles and have considerable potential as minimally-invasive biomarkers, since they are stable and some have been associated with disease phenotypes (Cheung et al., 2019). miRNAs are endogenous non-coding small RNA molecules that can be secreted into the circulation and exist in remarkably stable forms. Numerous efforts have been devoted to identifying suitable extracellular miRNAs for non-invasive biomarkers in cancer. However, not only technical challenges, but also cognitive challenges need to be overcome before practical application to be achieved. Cui et al. reviewed circulating miRNAs in terms of their biological function and basic transport carriers; extracellular cell communication process; roles as reliable cancer biomarkers and used in targeted cancer therapy; and challenges for clinical application.

Apart from blood, saliva is an easily accessible liquid biopsy. Based on gene set enrichment analysis, Yu et al. found that programmed death-ligand 1 (PD-L1) expression positively correlated with inflammation in periodontitis. They found that the levels of PD-L1 mRNA in salivary exosomes were higher in periodontitis patients than in controls. They claimed that this is the first protocol on isolating and detecting exosomal RNA from saliva of periodontitis patients. Their assay of exosomes-based PD-L1 mRNA in saliva has potential to distinguish periodontitis from the healthy, and the levels correlate with the severity/stage of periodontitis.

Recently, long non-coding RNAs (lncRNAs) have been reported to be highly implicated in the development and progression of breast cancer. Wu et al. reviewed that the majority of lncRNAs exert their roles through the regulation of invasion, migration, EMT, and metastasis process. Not only tumor progression, increasing evidence showed that lncRNAs play a role in chemotherapy resistance. A number of publications has reported the link between miRNA and lncRNAs. In this Research Topic, Li et al. demonstrated that lncRNA DLEU1 was upregulated in BCA tissues and high DLEU1 expression correlated with a poorer prognosis of BCA patients. The authors demonstrated that DLEU1 induced cell proliferation, invasion, and cisplatin resistance of BCA cells by de-repressing the expression of HS3ST3B1 through sponging miR-99b. Low miR-99b and high HS3ST3B1 levels were correlated with worse prognosis in patients with BCA. Ectopic expression of HS3ST3B1 or inhibition of miR-99b reversed DLEU1 knockdown mediated suppression of cell proliferation, invasion, and cisplatin resistance. They concluded a novel role for the DLEU1/miR-99b/HS3ST3B1 axis in regulating proliferation, invasion, and cisplatin resistance of BCA cells.

Recently, lncRNA has been shown as a competing endogenous RNA (ceRNA) to hinder miRNA functions that participate in posttranscriptional regulatory networks in tumors. However, few studies have focused on predicting cholangiocarcinoma (CCA) prognosis, and no systematic examination has been performed on the prognostic value of ceRNA in CCA. Long et al. performed a comprehensive comparison on RNA-sequencing and miRNA profiles from TCGA database. They further validated the mRNA expression levels in Gene Expression Omnibus. In the survival analysis, they found that 26 lncRNAs, 3 miRNAs, and 13 mRNAs were prognostic biomarkers for patients with CCA. They disclosed a better understanding of the ceRNA network involved in CCA biology that may improve CCA diagnosis and prognosis.

On the other hand, circular RNA (circRNA) is a novel class of lncRNA, resulting from pre-mRNA back splicing with a stable circular conformation that regulates various biological processes. The aberrant expression of circRNA and its impact on a number of cancers is still unclear. Nevertheless, Jamal et al. reviewed the updated functional roles of circRNAs in acute myeloid leukemia (AML). They also provided the mechanistic insights, discussed the challenges and opportunities associated with circRNA-based diagnostic and therapeutic development in AML.

Apart from circulating cell-free DNA which has been extensively studied, the detection and quantification of circulating cell-free RNA has gained great attention. Owing to the development of high-throughput transcriptomic analyses, numerous RNA splice variants and circRNAs have been found to be differentially expressed in tumor patients compared to controls. de Fraipont et al. reviewed the contribution of these RNA splice variants and circRNAs to tumor progression, dissemination, or drug response in preclinical models. The potential of circRNAs and mRNA splice variants as candidate biomarkers for the prognosis and the therapeutic response of NSCLC in liquid biopsies was well-discussed. We know that early detection is crucial for patient management, Prof Wong led a study which making use of custom designed colorectal cancer-associated targeted RNA-Seq panel on the detection of plasma mRNA in colorectal adenoma patients and normal healthy subjects. They found that GSK3A and RHOA in adenoma patients were significantly lower than those in normal healthy subjects (Xue et al., 2019).

Among the ncRNA, there is a class called small nucleolar RNAs (snoRNAs) which can be divided into C/D box and H/ACA box snoRNAs. Emerging evidence has demonstrated that the mutations and aberrant expression of snoRNAs involved in cell transformation, tumorigenesis, and metastasis. Actually snoRNAs may serve as biomarkers and potential therapeutic targets of cancer. Liang et al. reviewed the biogenesis and functions of snoRNAs, as well as the association of snoRNAs in different types of cancers and their potential roles in cancer diagnosis and therapy.

Some landmark translational researches help cancer patients in a remarkable manner, such as MammaPrint, Oncotype DX, etc. (Matikas et al., 2019). It is appealing to develop a multi-gene panel to predict the treatment response or patient prognosis. For example, there is a six-hypermethylated gene panel associated with poor survival in patients with nasopharyngeal carcinoma (NPC), demonstrating its potential usefulness as a prognostic biomarker to clinicians in NPC management (Jiang et al., 2015). In this RT, Liu et al. constructed a co-expression network to identify hub genes related to the International Staging System (ISS) stage of multiple myeloma (MM). Further analyses including functional analysis of hub genes, univariate Cox proportional hazard regression analysis as well as least absolute shrinkage and selection operator penalized Cox proportional hazards regression model were employed. The hub genes were further validated in the test set and an independent validation cohort. Finally, nine hub genes (HLA-DPB1, TOP2A, FABP5, CYP1B1, IGHM, FANCI, LYZ, HMGN5, and BEND6) were identified to build a 9-gene prognostic signature. They found that the relapsed MM and stage III MM was associated with a high risk score based on the signature. Further prospective clinical trials are warranted to test its clinical utility.

This RT will be published as an Ebook. We wish that all these efforts may enhance our understanding of the RNA world and provide some insight on its potential clinical utilization. RNA is diverse, dynamic, and does not work alone, future research directions may involve multifunctional biomolecular diagnostic markers and technology platforms. We envision a more comprehensive understanding of cancer biology by multi-omics approaches which represent distinct aspects of the central dogma of molecular biology (Cho, 2010; Paczkowska et al., 2020).

## Author Contributions

WC drafted the manuscript. LC and CW edited the manuscript. All authors approved the manuscript.

## Conflict of Interest

The authors declare that the research was conducted in the absence of any commercial or financial relationships that could be construed as a potential conflict of interest.
